# Sensory processing disorder: Perceptions on the clinical role of advanced psychiatric nurses

**DOI:** 10.4102/hsag.v24i0.1197

**Published:** 2019-09-23

**Authors:** Madelein Lang, Emmerentia du Plessis

**Affiliations:** 1 School of Nursing Science, North-West University, Potchefstroom, South Africa

**Keywords:** Sensory processing disorder, Clinical role, Advanced psychiatric nurse, Delphi technique, Clinical role description

## Abstract

**Background:**

No description of the clinical role of the advanced psychiatric nurse in the management of children with sensory processing disorder could be found for the South African context. This is a loss in clinical nursing practice with regard to diagnosis, treatment and research.

**Aim:**

To explore and clarify the perceptions of healthcare professionals in South Africa on the clinical role of the advanced psychiatric nurse in sensory processing disorder to provide a description of this role.

**Setting:**

The research was conducted by inviting registered healthcare professionals practicing in South Africa to complete several rounds of an on-line survey.

**Methods:**

An explorative and descriptive design was used. Purposive sampling was used to identify an initial sample of healthcare professionals, followed by snowball sampling. The Delphi technique was implemented with three sequential rounds, gathering data on the perceptions of the healthcare professionals regarding the clinical role of the advanced psychiatric nurse in sensory processing disorder.

**Results:**

The following main themes crystallised from the data – (1) Specialised training of the advanced psychiatric nurse (APN) on sensory processing disorder; (2) Interventions carried out by the APN with regard to sensory processing disorder; (3) Adequate support to the family with regard to sensory processing disorder; and (4) Referral of a child with sensory processing disorder.

**Conclusion:**

The study indicates that the healthcare professionals who are experts in delivering healthcare to children with sensory processing disorder agree that the APN with additional training in this condition has a clinical role to play in rendering healthcare to these healthcare users. A preliminary clinical role description could be formulated. Recommendations for nursing practice, nursing education and further research were formulated.

## Introduction and problem statement

Sensory processing disorder (SPD) is a neurological condition where the information received by the senses is not used effectively by the brain (Kranowitz 2005; Miller et al. 2011). This condition is a reality in the lives of many children. They are left to cope with the challenge SPD presents on a daily basis. According to Kranowitz (2006), sensory processing is a normal process that forms part of a human’s daily neurological functioning, and this process of neurological functioning cannot be observed objectively. The process of defining sensory processing on a more concrete and clinical level is, therefore, still happening on an ongoing basis (Alterio 2007), and how we make sense of our physical experiences on a sensory level is to a large extent still a mystery.

Historically, sensory integration (also referred to as sensory processing) theory and the treatment of conditions related to dysfunctions of the sensory integration system were first identified and developed by an occupational therapist, A. Jean Ayers (2005). Ayres laid the foundation for describing terminology specific to sensory integration and explaining what the basic theory is with regard to individuals battling with disorders related to sensory regulation and processing (Miller & Lane 2000).

Currently, SPD is used as the diagnostic term (label) by the majority of health professionals when referring to individuals experiencing intricacies in sensory processing that affect their daily functioning (Byrne 2009). The diagnosis and treatment of SPD are mainly reserved for the study field of occupational therapy and is a specialisation field at postgraduate level. The Mental Health Information Committee of the Children’s Hospital of Eastern Ontario (CHEO 2009) mentioned in an article on SPD that the knowledge of sensory-processing disorders does not form part of the professional knowledge of many doctors (Anon 2013). Doctors are of the opinion that there is a lack in evidenced-based research to support this condition as a differential diagnosis. It is also the academic experience of the researcher − during her studies as an advanced psychiatric nurse (APN) student − that specific education and training regarding SPD are not incorporated in the current undergraduate and postgraduate nursing programmes. This is a matter of concern in the sense that there are APNs working in primary healthcare settings in both the public and private sectors who may come into contact with patients presenting signs and symptoms of this condition. The fact that SPD is not currently included in formal nursing education of APNs can present a ‘lost opportunity in diagnosis, treatment, and research’ (Byrne 2009).

The clinical role of APNs entails promoting and maintaining the mental health of clients by implementing the nursing process (South African Nursing Council [SANC] 2014). Mendes, Da Cruz and Angelo (2014) have undertaken a critical literature analysis to conceptualise the clinical role of advanced clinical nurses. They have found that the clinical role of advanced clinical nurses is constantly evolving as advanced practices are characterised by continual changing of functions and designations. This finding stresses the importance of advanced clinical nurses – they are able and competent to develop professionally and to perform advanced psychiatric nursing care applicable to all mental health conditions and disorders, including SPD. However, studies have indicated that stakeholders do not clearly understand the clinical role of advanced clinical nurses, such as APNs (Kilpatrick et al. 2013).

With this need for role clarification of advanced nurse practitioners in mind, expert opinions of healthcare professionals were sought with regard to the perceived clinical role of APNs in the identification and management of children with SPD. These healthcare professionals know what the healthcare needs of children with SPD are and how their professional role may overlap with the clinical role of APNs. Healthcare professionals − as referred to in the context of this study − included educated individuals experienced in providing healthcare services to children diagnosed with SPD: paediatricians; paediatric psychiatrists; advanced practitioner nurses qualified in the field of community health, neonatal or psychiatric nursing; educational psychologists; occupational therapists; physiotherapists; speech therapists; and social workers.

## Purpose of the study

The purpose of the study was to explore and describe the perceptions of healthcare professionals who are involved in healthcare to children diagnosed with SPD with regard to the clinical role of APNs in relation to SPD. The study aimed to provide APNs and other healthcare professionals with a description of what the clinical role of APNs is when mental healthcare is provided to children presenting with signs and symptoms of SPD.

## Definitions of key concepts

### Sensory processing disorder

A condition of neurological origin, identified when incoming information to the brain − received via the senses − is not processed in an organised and accurate way (Kranowitz 2005), resulting in inaccurate processing and judgement of sensory information with regard to smell, touch, movement, sound and taste. Children can experience difficulty with moving, regulating emotions, attention problems and adapting responses (Kranowitz 2005).

### Advanced psychiatric nurses

Advanced psychiatric nurses are registered nurses registered with the SANC − and educationally prepared at a Master’s or doctorate level in the field of psychiatric and mental health nursing. Currently, the competencies of advanced nurse practitioners in psychiatry are still being clarified by subcommittees of the SANC. However, upon receiving their qualification they are registered as advanced professional nurses with the SANC as an additional qualification (Temane, Poggenpoel & Myburg 2014).

### Clinical role of advanced psychiatric nurses

This entails ‘the complex process of interaction between the nurse and the client’ (Mendes et al. 2014). It also entails promoting and maintaining the mental health of the clients of APNs by implementing the nursing process (ANA 2012). This process enables APNs to assess, diagnose and treat signs and symptoms that indicate mental health illness and psychiatric disorders. In this research study, the focus was on the clinical role of APNs in assessing, diagnosing and treating children with SPD.

## Research design

An explorative and descriptive design was applied in the research study. The Delphi technique was used as the chosen methodology. The Delphi design aimed to reach consensus on a topic among a group of experts or where existing opinions are not clear (Hanafin 2004). The Delphi technique was appropriate in this research where it was necessary to obtain the perceptions of healthcare professionals as experts on the clinical role of APNs in treating children with SPD.

## Research method

### Study population, sampling and setting

The population included registered healthcare professionals in South Africa who are experienced and knowledgeable in providing healthcare services to children with SPD. Paediatricians, paediatric psychiatrists, advanced nurse practitioners in the field of community health and neonatal or psychiatric nursing, educational psychologists, occupational therapists, physiotherapists, speech therapists and social workers were included. All the aforementioned health professionals, except nursing professionals (SANC registered), had to be registered with the Health Professions Council of South Africa (HPCSA), and they all had to be involved in the healthcare of children diagnosed with SPD for at least 3 years.

The sampling of the population initially entailed purposive sampling, followed by a snowball sampling strategy, as the researcher was not able to locate all the professionals in the population (Brink, Van der Walt & Van Rensburg 2012; Wagner, Kawulich & Garner 2012). The initial participants were identified from a contact list based on the researcher’s private practice experience in consulting with health professionals who provide healthcare to children with SPD. Electronic advertisements via email were sent to initial participants, inviting them to participate in the study. These initial participants were asked to identify and invite additional participants. The same participants who provided their informed consent were included for all the data-gathering rounds.

### Data collection and analysis

Data collection took place by implementing three data-gathering rounds. Each round was scheduled 2–3 weeks apart.

#### Round 1 data collection

The first round of data collection was less structured (Keeney et al. 2011) and was initiated by the researcher sending the participants an electronic open-ended question by making use of the SurveyMonkey software program (SurveyMonkey Inc. 2015).

The research question formulated to initiate the first round was:

According to the American Nurses Association (ANA 2012), the clinical role of advanced psychiatric nurse practitioners is defined as one of promoting and maintaining the mental health of the clients of advanced psychiatric nurses by implementing the nursing process. This process enables advanced psychiatric nurses to assess, diagnose and treat signs and symptoms that indicate mental health illness and psychiatric disorders. What do you think is the clinical role of advanced psychiatric nurses in delivering mental healthcare to children with sensory processing disorder? Please provide a role description as complete and comprehensive as you can.

A trial run was conducted to test the clarity and relevance of this question by inviting a nursing specialist who has extensive knowledge about SPD and practical experience in rendering healthcare to children to assess the clarity and relevance of the question.

18 participants agreed to informed consent included in the Survey Monkey program, thereby enrolling as participants and gaining access to the open-ended question.

#### Round 1 data analysis

The data gathered during Round 1 were analysed by making use of a thematic analysis (Creswell 2014; Wagner et al. 2012) to generate themes and statements relating to the clinical role of APNs in SPD. Tesch’s (Creswell 2014) eight steps were applied in coding the raw text data.

An independent co-coder experienced in qualitative research analysed the data. A consensus meeting was scheduled to reach consensus with regard to themes and sub-themes. The themes identified were described as specific statements on the clinical role of APNs in SPD (see the first column in Table 1 below).

**TABLE 1:** Statement consensus.

Statements	% 1–2 Consensus 51% or more		Interquartile range Consensus 2.5 or less		Standard deviation Consensus 1.5 or lower	
	Round 2	Round 3	Round 2	Round 3	Round 2	Round 3
**Theme A: Specialised training of APNs in SPD**						
(1) APNs must be able to identify the basic components of SPD	100.00	100.00	1.00	0.25	0.48	0.42
(2) It is vital for APNs to understand SPD	92.30	100.00	1.00	0.25	0.87	0.42
(3) APNs need additional training to recognise the signs and symptoms of SPD as early as possible	92.30	100.00	1.00	0.00	0.65	0.32
(4) SPD is a specialised field and additional training is, therefore, required for APNs	100.00	100.00	0.50	0.25	0.44	0.42
(5) APNs need to identify children who are at risk for SPD	100.00	100.00	0.50	1.00	0.44	0.48
(6) APNs need to be experienced in the field of SPD and its assessment	53.80	70.00	2.50	2.25	1.26	1.38
**Theme B: Interventions carried out by APNs with regard to SPD**						
(7) If APNs understand SPD, they can alter the handling and treatment of children	84.6	70.00	1.00	2.25	1.26	1.42
(8) The role of APNs includes the assessment, nursing diagnosis and treatment of SPD	**15.40**	**40.00**	2.00	2.25	1.44	1.29
(9) APNs must help to minimise the emotional trauma of children when exposed to a hospital environment	100.00	100.00	0.00	0.25	0.28	0.42
(10) APNs must implement sensory strategies in conjunction with the medical team	100.00	77.80	1.00	1.50	0.49	1.27
(11) APNs must provide a treatment programme to children and their families	**15.40**	**40.00**	2.00	2.25	1.28	1.33
(12) APNs must adapt to the environment that is within the control/abilities of APNs to support sensory organisation	100.00	100.00	1.00	1.00	0.52	0.53
**Theme C: Adequate support to families with regard to SPD**						
(13) APNs need to assist families in receiving comprehensive services available to them	100.00	100.00	0.50	0.25	0.44	0.42
(14) APNs need to adequately prepare parents emotionally and physically with regard to possible surgical interventions	100.00	90.00	1.00	1.00	0.52	1.20
(15) APNs must provide parent counselling and information regarding SPD	**46.20**	66.70	2.00	**3.00**	1.28	1.32
(16) APNs must assist families in carrying out the prescribed sensory interventions and home programmes	69.20	90.00	2.00	1.00	1.26	0.97
(17) APNs need to support and train parents regarding SPD	**30.80**	**40.00**	2.00	2.25	1.17	1.29
**Theme D: Referral of children with SPD**						
(18) APNs need to refer children to an occupational therapist for further management	100.00	100.00	0.00	0.00	0.28	0.32
(19) APNs need to refer to an appropriate professional to treat the children and their families	100.00	100.00	0.00	0.25	0.39	0.42
(20) APNs should facilitate communication between all the professionals working with the children	91.70	100.00	1.00	1.00	0.67	0.48

Note: Data not in bold text indicate consensus and bold text indicate non-consensus.

APN, advanced psychiatric nurse; SPD, sensory processing disorder.

Themes and sub-themes are discussed by referring to the consensus reached, the remarks and comments made by the participants and to relevant literature. Results from the different rounds of the Delphi are referred to as R 2 (Round 2) and R 3 (Round 3).

#### Round 2 data collection

The statements identified from Round 1 were grouped and listed under four main themes to create a statement questionnaire in the form of a 5-point Likert scale (strongly agree to strongly disagree) and space was made available to comment on answers and presented to the participants for Round 2 (Wagner et al. 2012). The original opinions of the participants were integrated into the statements so that they could identify their own ideas. The statement questionnaire was then sent electronically to the same participants who responded to Round 1 (*n* = 18). The software program Survey Monkey has a function that can control this aspect. The anonymity of the participants was, therefore, maintained. The focus of Round 2 was on gathering quantitative data on the consensus reached between experts on statements that describe the role of APNs in SPD, as derived from Round 1. Only 13 (*n* = 18) of the participants completed the questionnaire of Round 2 within the 7 working days allocated.

#### Round 2 data analysis

The data analysis of Round 2 included descriptive data with frequency distributions of the quantitative data and a thematic analysis (Creswell 2014) of the qualitative data. Tesch’s eight steps were used again (Creswell 2014). The researcher used a statistical software program (Mouton 2001; Wagner et al. 2012) known as the Statistical Package for the Social Science (SPSS) to analyse the quantitative data with the support of a statistical consultant. The consultant delivered descriptive statistics, frequency scores together with a percentage and mean for each statement. Consensus was determined by combining the following three measures (Giannarou & Zervas 2014):

- At least 51% of the participants must have responded to the category ‘strongly agree’, which lies between the values 1 and 2 on a 5-point Likert scale.
- The interquartile range must have been below 2.5.
- The standard deviation must have been below 1.5.

#### Round 3 data collection

After a data analysis of Round 2, descriptive data reflecting consensus were included in the same questionnaire used in the previous round and was sent to the participants to agree or disagree. By including the results of the consensus data and comments of the previous rounds, the participants were given the opportunity to re-evaluate their previous responses and alter responses if needed. Only 10 participants (*n* = 18) responded in Round 3.

#### Round 3 data analysis

The raw data of Round 3 were again sent to the statistician for a quantitative data analysis identical to the methods used with the descriptive data of Round 2. The Round 3 data analysis also entailed quantifying the degree of consensus reached in the form of percentages of agreement or disagreement (Giannarou & Zervas 2014) and a thematic analysis (Creswell 2014) for the motivation of answers, similar to the data analysis methods used in Round 2.

### Ethical considerations

The fair selection of participants was established by using inclusion and exclusion criteria that guided the selection process. An informed consent page was included and had to be agreed upon before the participants could proceed to the online questionnaire that was used during Round 1. The participants were given the right to privacy and anonymity by providing them with the opportunity to complete the online questionnaire anonymously. Their personal information was concealed by the Monkey Survey program. All agreements with the participants were honoured by the researcher, such as keeping to due dates, and the participants were made aware of the fact that they could withdraw from the study at any given time without consequences. The researcher gained ethical clearance from the Research Ethics Committee of the North-West University, Potchefstroom Campus (Reference NWU-00006-16-A1).

### Rigour

Trustworthiness was promoted by following the constructs of Guba, as referred to by Shenton (2004). By using the constructs of credibility, transferability, dependability and confirmability, the researcher ensured that the aspects of truth value, applicability, consistency and neutrality were achieved. The following strategies were implemented to promote rigour in this study: implementing a trial run; using an independent co-coder for data analyses; and conducting a literature control.

## Findings and discussion

Table 1 shows the results from Round 1 (four main themes and related statements), Round 2 and Round 3 (consensus data). These results include the quantitative and qualitative data. The numeric data areas coloured in regular and bold face indicate consensus and non-consensus, respectively.

### Theme A: Specialised training of advanced psychiatric nurses in sensory processing disorder

The top-ranking statements that the experts agreed on with regard to APNs having specialised training in SPD were that APNs must be able to identify the basic components of SPD (R 2 = 100% and R 3 = 100%); that SPD is a specialised field and additional training is required (R 2 = 100% and R 3 = 100%); and that APNs should identify children who are at risk for SPD (R 2 = 100% and R 3 = 100%). These findings reflect a strong agreement by the participants that APNs must indeed receive additional training in SPD to be able to identify children who are at risk for SPD by being able to identify the basic components of this disorder.

Advanced psychiatric nurses understanding SPD (R 2 = 92.3% and R 3 = 100%) and needing additional training in SPD to be able to recognise signs and symptoms as early as possible (R 2 = 92.3% and R 3 = 100%) were also viewed as statements that should be included in the theme of APNs needing specialised training in SPD. These findings are in line with available literature (Champagne & Stromberg 2004; Courtenay, Stenner & Carey 2010; Kranowitz 2005; McElhinney 2010). Currently, training in sensory integration is presented by the South African Institute for Sensory Integration (SASI), and health professions that can apply for this 5-year course are occupational therapists, physiotherapists and speech therapists. Once completed, these professionals can register as certified Ayres sensory integration therapists and are subsequently listed on the official website of SASI (www.instsi.co.za).

A further statement was that APNs should be experienced in the field of sensory development and the assessment thereof (R 2 = 53.8% and R 3 = 70%). Although the consensus of the participants of Round 2 was low (53.8%), consensus of the participants for Round 3 amounted to 70% − a strong indication that the panel of healthcare experts agree that the specialised training of APNs should include competency to assess sensory development. Several of the participants expressed their concern by commenting that APNs need to identify SPD and only intervene if trained.

A paper delivered by Byrne (2009), written to support the role of nurse practitioners with regard to children with SPD, stresses the fact that nurses are in a unique position to ‘fill the knowledge gaps in assessment, treatment, education and research’ with regard to rendering healthcare to children with SPD. Nursing literature further supports the clinical role of APNs in the domain of being able to perform evaluations or assessments by relying on ‘tools’, such as observation, enquiry, attentiveness and monitoring (Courtenay et al. 2010). Assessments by APNs include taking a systematic patient history followed by a full physical examination (Courtenay et al. 2010; Jones et al. 2010).

### Theme B: Interventions carried out by advanced psychiatric nurses with regard to sensory processing disorder

Three statements that were strongly agreed with were that APNs must help minimise the emotional trauma of children when exposed to a hospital environment (R 2 = 100% and R3 = 100%); APNs must implement sensory strategies in conjunction with the medical team (R 2 = 100% and R 3 = 77.8%) and must adapt to the environment that is within the control/abilities of APNs to support sensory organisation (R 2 = 100% and R 3 = 100%).

These statements are supported by the existing literature of nurses undertaking interventions to manage symptoms as they present (Aitken et al. 2008; Lofmark, Smide & Wikblad 2006). APNs are able to work closely with clinical providers (Hanrahan et al. 2011; Lammon, Stanton & Blakney 2010) and are, therefore, able to implement sensory strategies provided by the medical team.

If APNs understand SPD, they can alter the handling and treatment of children, and consensus was reached by the participants that this must be an intervention performed by APNs in treating SPD (R 2 = 84.6% and R 3 = 70%). Advanced nurse practitioners are trained in neurological assessment (McElhinney 2010) and serve as a foundation to support further training in the diagnosis and treatment of SPD.

Interestingly, the role of APNs that includes assessment, nursing diagnosis and treatment of SPD (R 2 = 15.4% and R 3 = 40%) and APNs being able to provide a treatment programme to children and their families (R 2 = 15.4% and R 3 = 40%) resulted in a strong non-consensus on a percentage level. The participants supported their disagreement by commenting that APNs are in need of additional training in sensory integration before any treatment can occur or referrals to qualified health professionals.

However, literature reveals that APNs can engage in the process of assessment and evaluation and that this process forms part of the clinical role of APNs (Roberts-Davis & Read 2001). The treatment regime planned by APNs may include interventions that are focused on managing the symptoms of the condition (O’Connor, Peters & Walsh 2008). It is, therefore, clear that the clinical role of APNs in SPD should include the attributes of being able to assess, diagnose and plan treatment for children presenting with SPD, while the results of this study illuminate the need for an awareness among healthcare professionals on the important role of APNs.

### Theme C: Adequate support to families regarding sensory processing disorder

Consensus was reached that APNs must provide adequate support to families with regard to SPD and make use of the comprehensive services available to them (R 2 = 100% and R 3 = 100%) and that APNs must adequately prepare parents emotionally and physically with regard to possible surgical interventions (R 2 = 100% and R 3 = 90%).

Following the systems approach (Withrow 2007), APNs are in the position to support families by involving them when decisions are made with regard to the best care for their children (Aitken et al. 2008). Establishing a trusting relationship with both patients and family members by showing appropriate compassion can enhance the role of APNs in rendering psychosocial care to patients and their families (O’Connor et al. 2008).

Assisting families in carrying out prescribed sensory interventions and home programmes was also agreed by the experts (R 2 = 69.2% and R 3 = 90%). Aitken et al. (2008) state that nurse practitioners should ‘engage in activities’ that will increase the level of practice and improve the outcome of healthcare rendered. However, some participants voiced their concern with regard to what the role of occupational therapists and APNs is when it comes to assisting families in carrying out sensory interventions and home programmes.

Research undertaken by Duner (2013) aimed at examining professional collaboration and boundaries among inter-professional care-planning teams by studying two different care teams. The outcome of the study revealed that these teams strived to deliver an integrated service to reach ‘holistic assessments’. However, there are certain preconditions (Duner 2013). Duner’s overall conclusion was that the roles of all professionals with regard to assessing needs and planning of care should be clarified. When focusing on implementing strategies to improve care outcome (Mendes et al. 2014), ANPs should review practice on a critical level (Aitken et al. 2008) and introduce changes gradually.

Advanced psychiatric nurses providing parent counselling and information regarding SPD was not regarded as applicable to the role of APNs providing support to families in Round 2, but in Round 3 the responses reached consensus (R 2 = 46.2% and R 3 = 66.7% with a mean of 56.4%). Participants commented that the occupational therapist should still be included, and parent counselling and information should not only be provided by the APN.

The participants felt that the support to and training of parents regarding SPD should not be included in the role of APNs (R 2 = 30.8% and R 3 = 40%). Motivated responses mostly focused on ‘to refer to a specialist’. According to the participants, providing counselling to parents and information about SPD and offering support by training parents are not applicable to the clinical role of APNs with regard to SPD. A clinical competency of APNs is the role of acting as information managers and educators (Tornabeni & Miller 2008). Another clinical competency of APNs is to provide counselling to patients and families (O’Conner et al. 2008). It is also the responsibility of APNs to be accurate and reliable (Lofmark et al. 2006) and being able to identify any limitations in their practice (Miles et al. 2007). APNs must have the ability to manage an evidence-based practice (Mendes et al. 2014) and by adhering to this, APNs are able to provide counselling and support to parents.

### Theme D: Referrals of children with sensory processing disorder

The highest-ranking statements were the need to refer children to occupational therapists for further treatment (R 2 = 100% and R 3 = 100%), and APNs must refer children to appropriate professionals to treat them and their families (R 2 = 100% and R 3 = 100%). Consensus was reached regarding APNs who must facilitate communication among all professionals working with children (R 2 = 91.7% and R 3 = 100%). This finding suggests that the participants were all in favour of APNs facilitating the process of communication among professionals working with children.

The professional commitment of APNs enables them to function as part of a healthcare team (Aitken et al. 2008; Mendes et al. 2014). It is important for APNs to be aware of the limitations of their practice (Miles et al. 2007) and must be able to refer to other health professionals, according to a need’s assessment (Pulcini et al. 2010).

### Limitations

The small sample size (*n* = 18) and the fact that the majority of the health professionals who participated in the study were occupational therapists (*n* = 15) are two strong indicators that the results of this study cannot be generalised to the rest of healthcare professionals in South Africa. After Round 1 (data analysis), it was evident that the participants were not fully aware of what the professional practice of APNs entails. However, they were able to provide their opinions on what the role of APNs in caring for children with SPD should be.

### Recommendations

Supported by the findings and conclusions of this study, the following recommendations seem appropriate to nursing practice, research and education.

#### Nursing practice

The clinical role of APNs in SPD is still a grey area with regard to role description. The consensus reached in this study by the participants concerning specific competencies does reflect on the clinical role description for advanced nursing practice in SPD. Advanced psychiatric nurses should, therefore, define professional practice boundaries by integrating the statements as agreed on and must attend advanced training courses in SPD. Advanced psychiatric nurses with specialised training in SPD can act as supervisors or consultants to other nurse practitioners in this regard. In-service training workshops by specially trained APNs can also be presented to the nursing personnel of hospitals and clinics to inform them about SPD and the management of this condition within a hospital and primary healthcare environment. Lastly, the researcher compared the results of the study to the generic competency framework available for advanced nurse practitioners (SANC 2014) and found it possible to integrate the clinical role of APNs into this framework (Table 2). This framework can be used during in-service training and workshops to continue the discussion on the clinical role of the advanced psychiatric nurse in providing care to children with SPD.

**TABLE 2:** Summary of the clinical role description of advanced psychiatric nurses concerning sensory processing disorder. Care provision and management of children with SPD by APNs.

Domain	Core competencies
**Health promotion**	APNs: must provide parent counselling and information regarding SPD need to support and train parents regarding SPD
**Assessment**	
This includes a systematic health and nursing assessment of both objective and subjective data specific to the speciality practice area. It also includes ordering and the interpretation of diagnostic tests, according to the scope of practice for advanced nursing practice to formulate a nursing diagnosis that will guide the care plan. The information gathered should be documented and shared in a timely manner in line with nursing practice standards and institutional policies.	APNs must be able to identify the basic components of SPD It is vital for APNs to understand SPD APNs need to: identify children who are at risk for SPD be experienced in the field of sensory development and its assessment
**Planning**	
The formulation of a comprehensive care plan that is specific to the individual by applying critical thinking and reasoning skills that are grounded in advanced nursing knowledge, which includes other disciplines. Collaborating with the health team when setting priorities and when revising the care plan. Documentation of the process of planning for healthcare is of key importance.	If APNs understand SPD, they can alter handling and treatment APNs must provide a treatment programme to children and their families
**Implementation**	
Implementing advanced procedures, treatments and interventions based on the care plan and best practice standards and documenting these interventions in a timely manner.	APNs: must implement sensory strategies in conjunction with the relevant medical team Adaptation of the environment that is within the control/ability of APNs is necessary to support sensory organisation APNs need to: assist families in accessing comprehensive services available to them adequately prepare parents emotionally and physically with regard to possible surgical interventions APNs must provide parent counselling and information regarding SPD APNs need to: assist families in carrying out the prescribed sensory interventions and home programmes support and train parents regarding SPD refer children to occupational therapists for further management refer patients to appropriate professionals to treat the children and their families APNs should facilitate communication among all the professionals working with children
**Evaluation**	
Monitoring and documenting progress of expected outcomes by evaluating them in collaboration with healthcare users, families or caregivers and health teams, and then using the evaluation data to alter the care plan if needed. If a gap is identified in the healthcare provided by other team members, support should be offered to those members.	APNs should facilitate communication among all of the professionals working with children
**Therapeutic communication and relationships**	
Therapeutic relationships should be initiated and developed until they are terminated by making use of advanced communication and interpersonal skills. Keep relationship boundaries with healthcare users in place by being respectful and unbiased. Provide clear, consistent and accurate information by using communication skills. Facilitate access to information and refer to most appropriate person if needed. Maintain open communication with healthcare teams by sharing information regarding the views of healthcare users and their families in a confidential manner.	APNs must help to minimise the emotional trauma of children when exposed to a hospital Adaptation of the environment that is within the control/abilities of APNs is necessary to support sensory organisation APNs need to adequately prepare parents emotionally and physically with regard to possible surgical interventions APNs must also provide parent counselling and information regarding SPD APNs need to: support and train parents regarding SPD refer children to occupational therapists for further management refer to appropriate professionals to treat the children and their families APNs should facilitate communication among all of the professionals working with children

APN, advanced psychiatric nurse; SPD, sensory processing disorder.

#### Nursing research

Future research on refining the clinical role of APNs in sensory processing is needed. Research can be done with APNs in private practice to explore their current knowledge about SPD and their need for advanced training in this field of expertise.

#### Nursing education

This study recommends the development of an advanced training programme in SPD and the management thereof for APNs. Advanced psychiatric nurses are trained in childhood illnesses regarding mental health and they rely on this knowledge base to assess, diagnose and treat psychiatric conditions. Although SPD is not included in the DSM V, it is suggested that theoretical information on SPD and the treatment thereof must be included in the advanced psychiatric nursing curricula.

## Conclusion

The results of the study revealed that the participants who are experts in SPD are of the opinion that APNs must receive advanced or specialised training to render mental healthcare to children with SPD. The clinical role of APNs with advanced training in SPD entails, therefore, that they should have an understanding of this condition with regard to the basic concepts thereof. Their clinical role further prescribes the identification of signs and symptoms of SPD as early as possible and, if needed, referrals of children to the most appropriate health professionals. Adequate support to families is also a possibility − APNs can assist families in receiving comprehensive services, can support them emotionally and physically for when surgical interventions are needed and can assist them with applying prescribed home programmes.

The notion that APNs must be competent in assessing, diagnosing and treating SPD and being able to counsel parents regarding SPD was not favoured by the participants. The participants view these aspects as part of the role of occupational therapists trained in sensory integration. However, nursing literature reveals that the competency of assessments, diagnoses and planning treatment plans that include counselling and providing health education stands at the core of being able to practise nursing at an advanced level. There is a greater need for APNs to further improve their professional practice by committing to further education due to the fact that SPD is not included in nursing curricula.
